# Use of Adaptive Conjoint Analysis–Based Values Clarification in a Patient Decision Aid Is Not Associated with Better Perceived Values Clarity or Reduced Decisional Conflict but Enhances Values Congruence

**DOI:** 10.1177/0272989X241298630

**Published:** 2024-11-18

**Authors:** Nida Gizem Yılmaz, Arwen H. Pieterse, Danielle R. M. Timmermans, Annemarie Becker, Birgit Witte-Lissenberg, Olga C. Damman

**Affiliations:** Department of Communication Science, Amsterdam School of Communication Research/ASCoR, University of Amsterdam, Amsterdam, the Netherlands; Biomedical Data Sciences, Leiden University Medical Center, Leiden, the Netherlands; Department of Public and Occupational Health, Amsterdam Public Health research institute, Amsterdam UMC, Vrije Universiteit Amsterdam, Amsterdam, the Netherlands; Department of Pulmonary Diseases, Amsterdam UMC, VU University Medical Center, Amsterdam, the Netherlands; Department of Epidemiology and Biostatistics, Amsterdam UMC, Vrije Universiteit Amsterdam, Amsterdam, The Netherlands; Department of Public and Occupational Health, Amsterdam Public Health research institute, Amsterdam UMC, Vrije Universiteit Amsterdam, Amsterdam, the Netherlands

**Keywords:** values clarification methods, conjoint analysis, patient decision aids, decisional conflict, informed decision-making

## Abstract

**Background:**

Evidence is lacking on the most effective values clarification methods (VCMs) in patient decision aids (PtDAs). We tested the effects of an adaptive conjoint analysis (ACA)–based VCM compared with a ranking-based VCM and no VCM on several decision-related outcomes, with the decisional conflict and its subscale “perceived values clarity” as primary outcomes.

**Design:**

Online experimental study with 3 conditions: no VCM versus ranking-based VCM versus *ACA*-based VCM (*N* = 282; *M_age_* = 63.11 y, *s* = 12.12), with the latter 2 conditions including attributes important for a lung cancer treatment decision. We assessed 1) decisional conflict, 2) perceived values clarity (decisional conflict subscale), 3) perceived cognitive load, 4) anticipated regret, 5) ambivalence, 6) preparedness for decision making, 7) hypothetical treatment preference, and 8) values congruence (proxy). We performed analysis of covariance and linear regression. Age and level of deliberation were included as potential moderators, and we controlled for subjective numeracy (covariate). We exploratively tested the moderating effects of subjective numeracy and health literacy (without covariates).

**Results:**

We found no significant effect of type of VCM on overall decisional conflict or perceived values clarity. Age had a moderating effect: in younger participants, no VCM (v. ranking-based VCM) led to more values clarity, while in older participants, a ranking-based VCM (v. no VCM) led to more values clarity. Completing the ACA-based VCM, compared with no VCM, resulted in more values congruence.

**Limitations:**

The hypothetical choice situation might have induced lower levels of cognitive/affective involvement in the decision.

**Conclusions:**

This study found mixed effects of an ACA-based VCM. It did not decrease decisional conflict or increase perceived values clarity, yet it did improve values congruence.

**Implications:**

Completion of an ACA-based VCM in a PtDA may increase values congruence.

**Highlights:**

- An adaptive conjoint analysis or a ranking-based values clarification method did not decrease analog patients’ decisional conflict nor did it increase their perceived values clarity.
- In younger participants, no VCM (v. ranking-based VCM) led to more values clarity, while in older participants, a ranking-based VCM (v. no VCM) led to more values clarity.
- An adaptive conjoint analysis task for values clarification resulted in more values congruence.

To support patients in clarifying what is most important to them in treatment decision making, patient decision aids (PtDAs) often provide values clarification methods (VCMs).^1,2^ VCMs have been shown to help patients think about how desirable they consider characteristics of treatment options, to determine which they prefer, and to make more values-congruent choices.^1–4^

Rating and ranking exercises are common types of VCMs^5,6^ and are generally based on the assumption that patients are willing and able to deliberately evaluate and weigh characteristics of options (i.e., attributes). Rating exercises do not require tradeoffs between attributes; patients can attach equal importance to all attributes, and therefore, they likely do not provide much insight into order of importance. It has been shown that how patients fill in such rating scales does not always predict their actual health care choices.^7^ Ranking methods, alternatively, can provide more feedback on what matters most, but it remains questionable to what extent they contribute to values clarity and help in determining preferences. In longer lists of attributes, it is known that people rank attributes adequately at the top and at the bottom, but the middle part of the ranking is more unreliable.^8^ Moreover, ranking methods possibly require cognitive effort.^9^

A VCM based on conjoint analysis (CA) has been suggested to be an alternative to rating and ranking types of VCMs.^1,10^ CA is based on the assumption that a service or good, in this case a treatment option, can be broken down to its attributes and that the degree to which the service/good is valued depends on the levels of these attributes. CA is a technique that allows people to judge (hypothetical) cases in the form of combinations of attributes clustered at a particular level (i.e., vignettes). Based on how participants react to such vignettes (e.g., a choice between 2 vignettes or a rating of how much one leans towards one or the other vignette), preferences associated with the attributes (expressed in utilities), and ultimately the preference for a service/good, can be estimated.^11,12^ CA exists in different forms. In this study, we focus on adaptive CA (ACA), in which *adaptive* means that the CA task is customized to the individual, based on his/her previous answers, which then determine subsequent questions.^13^ Note that ACA is based partly on a different theoretical foundation than discrete choice experiments (DCEs).^14^ Also, DCEs require participants to choose between 2 options, while in ACA, participants usually indicate their preference strength on a scale.

CA-based values clarification is expected to depend for a larger part on intuitive or holistic evaluation, since it invites people to consider options, rather than separate attributes. It is known that people tend to rely for a great part on heuristics when they complete a CA task.^15,16^ While people may review options in detail at the beginning of a CA task, they have been shown to proceed to holistic evaluations during the task, that is, comparing attribute combinations instead of single attributes.^15^ Holistic evaluation of options based on considering multiple aspects of options may facilitate values clarification better than rating and ranking methods do. Witteman et al.^1^ suggested that comparative effectiveness research is needed to gain evidence about the superiority of different VCM tasks, including ACA.

VCM tasks that induce holistic evaluations may especially be suitable for older patients,^17–19^ those who deliberate less, lower numerates,^20,21^ and lower health literates.^22,23^ People who are older or who have lower levels of numeracy or health literacy likely have more difficulty processing information in an analytic way and are more prone to cognitive overload.^18,19,24^ Considering options more holistically, for instance in a CA-based VCM, may prevent overload and may thereby facilitate attention to relevant attributes. It is plausible that in people who deliberate more during the decision task, a CA-based or ranking-based VCM may not be of additional benefit, because these people are already thinking thoroughly about the best decision for them. However, for people who deliberate less during the task, a CA-based or ranking-based VCM may still stimulate a more thorough decision-making process, ultimately influencing outcomes.

The current literature is mixed on the effects of CA-based VCMs,^1^ most likely as a result of differences in study context, setting, and outcomes. One study on preferences in colorectal cancer screening showed that a CA-based VCM, compared with a rating- and ranking-based VCM, had no effect on perceived values clarity, an important aim of completing a VCM.^25^ Another study, however, showed in a pre-post design that a CA-based VCM decreased decisional conflict regarding surgical management of urethral stricture disease.^26^ In yet other studies, *a CA-*based VCM was associated with less decisional regret, more preparedness for decision making, and less decisional conflict compared with no VCM.^27–31^

Witteman et al.^1^ suggested that 4 key outcomes should be studied in comparative VCM-effectiveness research: decisional conflict, decision or decision intention, values congruence, and decisional regret. One key subscale of decisional conflict is perceived values clarity, in other words, the subjective feeling that one is clear in what matters to him/her in weighing the benefits and harms of options. Other key experiences of going through a VCM, which have been studied less, are perceived cognitive load, feelings of ambivalence, and anticipated decisional regret. Use of a VCM is generally thought to result in more values clarity, yet one can easily imagine that completing a VCM—of any type—can be cognitively burdensome and might result in ambivalence or decisional conflict, especially if multiple attributes appear important. How an ACA-based VCM influences such experiences compared with more traditional VCMs remains unknown. Moreover, how an ACA-based VCM works out for people who differ in information-processing skills (e.g., older v. younger people) and decision-making processes (e.g., deliberative v. intuitive process while completing the task), has not yet been studied.

This study compared an ACA-based VCM to a ranking-based VCM and no VCM, on several decision-related outcomes, and assessed potential moderating effects of age, level of deliberation, and exploratively subjective numeracy and health literacy. Table 1 presents the hypotheses and research questions.

**Table 1:** Overview of the Hypotheses and Research Questions

**H1:** Those exposed to a values clarification method (VCM) based on conjoint analysis and those exposed to a VCM based on ranking will experience more values clarity and less overall decisional conflict than those exposed to no VCM. **H2:** Those exposed to a VCM based on conjoint analysis will experience more values clarity and less overall decisional conflict than those exposed to a VCM based on ranking. **RQ1:** Are the effects of the manipulations (H1 and H2; VCM based on conjoint analysis, VCM based on ranking) on perceived values clarity and overall decisional conflict different for older patients compared with younger patients? **RQ2:** Are the effects of the manipulations (H1 and H2; VCM based on conjoint analysis, VCM based on ranking) on perceived values clarity and overall decisional conflict different for patients who deliberate less compared with patients who deliberate more? **RQ3:** What is the effect of the manipulation (VCM based on conjoint analysis, VCM based on ranking) on our secondary outcomes as specified below? • Perceived cognitive load • Anticipated regret • Ambivalence regarding treatment options • Preparedness for decision making • Hypothetical treatment preference **RQ4:** Are the effects of the manipulations (RQ3; VCM based on conjoint analysis, VCM based on ranking) on the secondary outcomes different for older patients compared to younger patients? **RQ5:** Are the effects of the manipulations (RQ3; VCM based on conjoint analysis, VCM based on ranking) on the secondary outcomes different for patients who deliberate less compared to patients who deliberate more? **Exploratory analyses:** • The testing of moderation effects (RQ1/RQ2 and RQ4/RQ5) was repeated with health literacy and subjective numeracy. • Additional analyses for RQ3/RQ4/RQ5 were performed with a proxy for values congruence.

a Please note that these exploratory analyses were not preregistered but rather prompted by a demonstrated effect of type of VCM on treatment preference and more recent literature on VCMs (Witteman et al.^1^).

## Methods

### Design

This study entailed an online posttest-only experimental design with 3 experimental conditions: no VCM versus ranking-based VCM versus ACA-based VCM. Participants were randomly assigned to one of the conditions. The Medical Ethics Committee of Amsterdam UMC, location VUmc, approved the study (2016.587). The study was preregistered (see https://osf.io/9y8vq/). Written consent was obtained from the participants.

### Participants

Eligible participants were adults aged 18 y or older who had (a history of) cancer other than lung cancer and mastered the Dutch language sufficiently (as registered by the research panel). Lung cancer patients and survivors were not included because they were expected to have already made a treatment choice and to have an initial treatment preference. Participants were categorized into younger (i.e., 18–64 y old) or older (i.e., ≥65 y). Participants were recruited through an online research panel (Flycatcher; ISO20252/26362 certified). An a priori sample size calculation was performed in G*Power for a 3 × 2 analysis of variance (ANOVA) with a small to medium effect size of 0.30 (Cohen’s *f*) and a 2-sided significance level of α = 0.05. This 3 × 2 design was used in light of RQ1 and RQ2 to allow for comparisons between 2 groups of participants. The sample size calculation showed that we needed to recruit at least 281 participants for sound power (0.95). We included 282 participants.

### Materials

In each condition, participants were presented with an introductory text and then exposed to a prototype PtDA concerning lung cancer treatment. The prototype consisted of core decision-relevant information (identical across experimental conditions) and 1 of 2 VCMs in the experimental conditions. (The stimulus materials are given in Appendix A.)

In the introductory text, participants were asked to imagine that they had recently been diagnosed with early-stage non-small-cell lung cancer (NSCLC) and that their physician had told them that they could choose between 2 treatment options with about equal chances of survival^i^: surgery versus stereotactic ablative radiotherapy (SABR). A very brief description of early-stage NSCLC, surgery and SABR was provided. Next, participants were asked to carefully go through the prototype PtDA and to use the information provided to make a hypothetical treatment preference.

The prototype was based on information from an existing PtDA (Amsterdam UMC Locatie AMC, Keuzehulp Longkanker). The prototype was specifically designed for this study to allow participants to review core decision-relevant information and to use different VCM versions. It described the following information: expected outcomes of 2 treatment options (e.g., survival rates), the treatment procedures (e.g., short-term effects on daily activities), and possible side effects and complications (e.g., mild versus severe side effects). Attributes, levels, and wordings were determined based on consensus meetings with medical professionals, patients, and researchers who had developed the existing PtDA and the clinical experience and scientific work of the pulmonologist and radiation oncologist involved.

**Figure 1:**
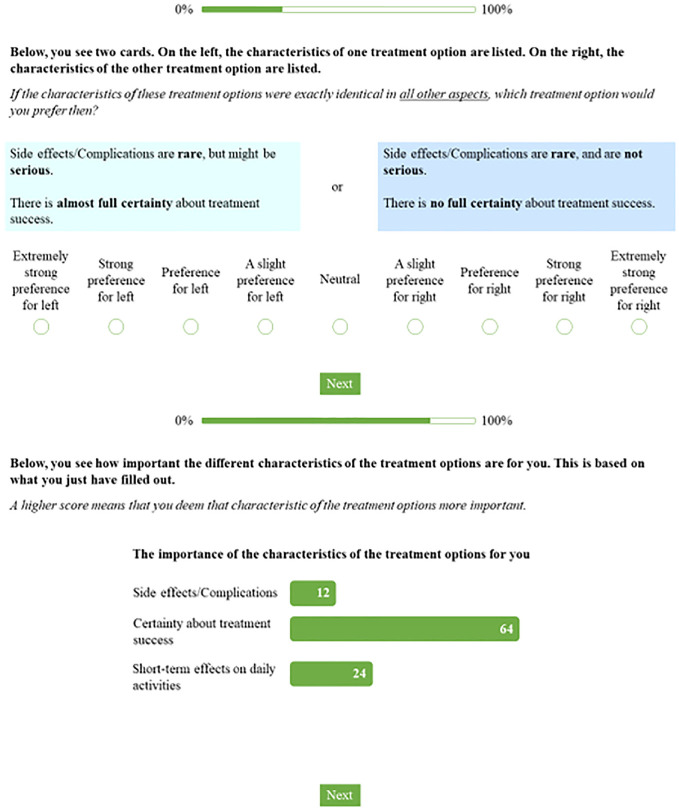
Screenshots of the conjoint analysis-based values clarification method. The upper screenshot is 1 example out of the 12 pairs of vignettes. The bottom screenshot shows what the instant feedback to participants looked like.

In the ranking-based VCM condition, participants were asked to rank 6 different attributes of treatments from “most important to me” to “least important to me.” The attribute list can be found in Appendix A. Next, participants were directed to the survey. In the ACA-based VCM condition, participants were exposed to 12 pairs of vignettes. Each vignette represented one treatment option consisting of combinations of attributes. For example, one vignette showed that treatment A is associated with multiple (severe) side effects and high certainty of treatment success (i.e., the extent to which it is certain that the cancer cells are gone after treatment) and another vignette showed that treatment B is associated with (almost) no side effects and low certainty of treatment success. The adaptive character of the task ensured that the (combination of) attributes in the vignettes were nearly equal in value to an individual participant, based on the participant’s earlier answers, thereby minimizing the number of comparisons needed. Per pair of vignettes, shown left and right on the screen, participants could indicate their relative preference (i.e., treatment option) on a 9-point scale (“extremely strong preference for left” to “extremely strong preference for right”). The vignette on the left represented SABR, and the vignette on the right represented surgery, but treatment labels were not shown to participants. After exposure to the 12 vignettes, participants were presented with instant individual feedback on what attributes of the treatment options (e.g., “Side effects/complications,”“Certainty about treatment success,”“Short-term effects of treatment on daily activities”) were most important to them, based on their answers (Figure 1). No feedback was provided on how the outcome of the ACA task aligned with one or the other treatment. The ACA task was programmed using Sawtooth Software (https://sawtoothsoftware.com/)

### Procedure

The research panel provided participants with a link to the online survey. Participants first read information about the study aim, data confidentiality, and voluntary participation. After providing informed consent, participants were randomly assigned to 1 of the 3 conditions. They were then asked to go through the prototype PtDA, including the VCM (ranking based or ACA based) or not, depending on experimental condition. Next, participants were directed to a survey, which was identical across conditions. In the 2 VCM conditions, participants could proceed to the survey only if they had gone through the entire VCM. Participants in the control group were asked to complete the ranking-based VCM after filling in the survey, to collect information on their values. Appendix B shows the ranking of attributes in all experimental conditions.

The research panel checked data quality based on responses to open questions, consistency in answers, straight lining, and time spent to complete the survey.

### Measures

Where relevant, items used per variable are presented in Appendix C.

#### Primary outcome variables

##### Decisional conflict

The 16-item Decisional Conflict Scale^34^ was measured on a 6-point Likert scale (0 = *yes* to 4 = *no*). To calculate the total score, we divided the sum of all items by 16 and then multiplied it by 25 (range scores, 0–100). The internal consistency of this measure was good (Cronbach α = 0.90). We took the mean to calculate a mean decisional conflict score. Higher scores demonstrated higher decisional conflict.

##### Perceived values clarity

The 3-item Values Clarity subscale of the Decisional Conflict Scale was measured. Internal consistency of the subscale was good (Cronbach α = 0.86). To calculate the total values clarity score, we reverse-coded the subscale so that higher scores represented higher values clarity and then divided the sum of the 3 items by 3 and multiplied it with 25. We took the mean to calculate a mean perceived values clarity score (range scores, 0–100).

#### Secondary outcome variables

##### Perceived cognitive load

Perceived cognitive load caused by the total information package was calculated as the mean of the scores on 4 items, each measured on a 5-point Likert scale (0 = *yes* to 4 = *no*).^35^ The internal consistency of the scale was good (Cronbach’s α = 0.89). Higher scores demonstrated higher perceived cognitive load (range scores, 0–16).

##### Anticipated regret

The anticipated regret was evaluated as a single item using a visual analog scale (VAS)^36^: “Sometimes people regret their treatment choice. You possibly thought about regretting your treatment choice as well. To what extent did regret play a role in your treatment choice?” (range scores, 0–10). Higher scores demonstrated higher anticipated regret.

##### Ambivalence towards treatment

Four items were measured on a 4-point Likert scale (0 = *not positive/negative at all* to 3 = *very positive/negative*).^37^ We calculated ambivalence by subtracting the absolute value of the difference between positive (P) and negative (N) attributes: (P + N)/2 − | P − N |. Scores around zero demonstrated less ambivalence; scores deviating more from zero demonstrated more ambivalence.

##### Preparedness for decision making

Preparedness for decision making was calculated as the mean of 6 of originally 10 items, measured on a 5-point Likert scale (0 = *not at all* to 4 = *a great deal*)^38^; we excluded items about interaction with health care professionals (range scores, 0–40). Internal consistency of the scale was good (Cronbach α = 0.88).

##### Hypothetical treatment preference

Hypothetical treatment preference was evaluated as a single item using a VAS scale: “Imagine that you would need to choose a treatment now. Which treatment option would you prefer?” (range scores, 0–10). Higher scores demonstrated a higher preference for SABR; lower scores demonstrated a higher preference for surgery.

##### Values congruence

We composed a dichotomous proxy outcome for values congruence after data collection (0 = values incongruent v. 1 = values congruent). This was not planned a priori and was prompted by literature on values clarification that was published after we had designed our study. For all participants, first, an “expected treatment preference” was constructed. In the no VCM and ranking-based VCM conditions, the most important attribute (i.e., ranked number 1) was considered to reflect people’s expected preference toward either surgery or SABR. If the attribute “the chances of survival are high” was ranked first, the second most important attribute (i.e., ranked number 2) was taken. See Appendix D for a more detailed description. In the CA-based VCM condition, the attribute with the highest expected utility score was considered to represent the expected treatment preference. Once an expected treatment preference was constructed for all participants, a new variable representing values congruence was constructed. If the expected preference and hypothetical treatment preference were aligned, this was labeled as “values congruent (1).” If the expected preference and hypothetical treatment preference were not aligned, this was labeled as “values incongruent (0).”

#### Demographics

We registered participants’ gender, age, level of education, prior medical knowledge, history of cancer diagnosis, and history of cancer treatment.

#### Moderators

Moderators of interest were age and deliberation, both continuous variables. Deliberation was calculated as the mean of 7 items, measured on a 5-point Likert scale (1 = *totally disagree* to 5 = *totally agree*; range scores, 7–35)^39^ (Cronbach’s α = 0.88). The measure is a process-related measure capturing to what extent participants deliberate (i.e., analytically weighed the pros and cons of options) while completing the current decision task, with higher scores suggesting a more analytic and thorough decision-making process.

#### A priori selected covariates

The a priori selected covariates were health literacy and subjective numeracy, both continuous variables, and measured with self-report instruments that capture the concepts subjectively.

Health literacy is the mean of 3 items of the Set of Brief Screening Questions, measured on a 5-point Likert scale (1 = *never* to 5 = *always*; range scores, 3–15)^40,41^ (Cronbach’s α = 0.51).

Subjective numeracy is the mean of the 8-item Subjective Numeracy Scale, measured on a 6-point Likert scale (i.e., 1 = *not at all good* to 6 = *extremely good*; range scores, 8–48)^42^ (Cronbach’s α = 0.70). Subjective numeracy is a valid estimation of actual numeracy.^42,43^

Health literacy and subjective numeracy were positively and significantly correlated, *r* = 0.33, *P* < 0.001. We planned to include either health literacy or subjective numeracy as covariate (see preregistration), depending on their respective association with age. Subjective numeracy showed a slightly stronger correlation with age (*r* = 0.05) than health literacy (*r* = 0.04) and was therefore selected.

#### Manipulation check

We performed a manipulation check to determine whether the ranking-based and ACA-based VCM conditions were sufficiently different in terms of their core difference: ranking individual attributes (analytic approach) versus comparing options (holistic approach). Two self-constructed items were used with a 5-point Likert scale (0 = *totally disagree* to 4 = *totally agree*): 1) “During the exercise, I had to rank the consequences of the treatment options from ‘most important to me’ to ‘least important to me’.” and (2) “During the exercise, I had to weight the 2 treatment options.” We calculated the mean score.

Participants in the ranking-based VCM condition perceived the VCM more as an exercise requiring to rank consequences of treatment options ( $\bar{x}$ = 4.35, *s* = 0.93) than to compare treatment options as a whole ( $\bar{x}$ = 3.70, *s* = 1.11), *t*(181)= 4.27, *P* < 0.001, 95% confidence interval (CI) [0.35; 0.94]. Participants in the ACA-based VCM condition perceived the VCM more as an exercise required to compare treatment options as a whole ( $\bar{x}$ = 4.39, *s* = 0.73) than to rank consequences of treatment options ( $\bar{x}$ = 3.96, *s* = 1.13), *t*(162.73) = −3.07, *P* = 0.003, 95% CI [−0.70; −0.15]. Hence, the manipulation was considered to be successful.

### Analysis

SPSS version 25 was used for data analysis. For the main analyses, several 2-way analyses of covariance were performed with ‘type of VCM” as independent variable, the primary and secondary outcome variables as dependent variable, age and deliberation as moderators (i.e., interaction terms), and subjective numeracy as covariate. Logistic regression analysis was performed for the dependent proxy-variable values congruence. A Bonferroni post hoc test was applied to adjust for multiple hypothesis testing. Table 2 describes the sample characteristics.

**Table 2:** Sample Characteristics

	No VCM (*n* = 99)	Ranking-Based VCM (*n* = 95)	ACA-Based VCM (*n* = 88)	Total (*N* = 282)
Demographics				
Gender (% female)	50.5	62.1	55.7	56.0
Level of education (%)^a^				
Low	13.1	29.5	29.5	23.8
Moderate	42.4	42.1	31.8	39.0
High	44.4	28.4	38.6	37.2
Cancer diagnosis (%)				
Breast	18.2	35.8	23.9	25.9
Urological	20.2	14.7	17.0	17.4
Skin	31.3	14.7	26.1	24.1
Other	15.2	13.7	12.5	13.8
Remaining diagnoses^b^	15.1	21.1	20.5	18.8
Treatment				
Surgery	39.4	28.4	51.1	39.4
Radiation therapy	21.2	28.4	20.5	23.4
Hormone therapy	13.1	12.6	11.4	12.4
Remaining treatments^c^	26.3	30.6	17.0	24.8
Radiation therapy as primary or secondary treatment (% primary)	38.3	26.7	19.4	28.7
Outcome variables ( $\bar{x}$ ±*s*)				
Decisional conflict (α = 0.90)	20.22 ± 14.59	20.69 ± 14.37	24.63 *±* 13.57	21.75 *±* 14.29
Perceived values clarity (α = 0.86)	81.57 *±* 18.57	84.74 *±* 16.43	80.78 *±* 18.02	82.39 *±* 17.72
Anticipated regret	3.58 *±* 2.15	3.54 *±* 2.21	3.24 *±* 2.03	3.46 *±* 2.13
Cognitive load (α = 0.89)	5.46 *±* 2.48	5.64 *±* 2.49	5.85 *±* 2.07	5.65 *±* 2.36
Preparedness for DM (α = 0.88)	25.18 *±* 3.51	25.18 *±* 3.52	24.36 *±* 3.98	24.93 *±* 3.67
Ambivalence ( $\bar{x}$ ±*s*)				
Surgery	1.36 *±* 0.91	1.37 *±* 0.90	1.32 *±* 1.00	1.35 *±* 0.93
SABR	1.28 *±* 0.81	1.39 *±* 1.00	1.40 *±* 1.05	1.35 *±* 0.95
Hypothetical choice	6.28 *±* 3.02^b^	6.07 *±* 2.99 ^c^	5.00 *±* 3.00^d,e^	5.81 *±* 3.05
Moderators ( $\bar{x}$ ±*s*)				
Age, y	63.23 *±* 13.44	62.31 *±* 11.69	63.84 *±* 11.04	63.11 *±* 12.12
Deliberation (α = 0.88)	30.37 *±* 4.32	30.79 *±* 4.25	30.52 *±* 4.07	30.56 *±* 4.21
Covariates ( $\bar{x}$ ±*s*)				
Health literacy (α = 0.51)	12.71 *±* 1.83	12.49 *±* 2.04	12.69 *±* 1.98	12.63 *±* 1.94
Subjective numeracy (α = 0.70)	35.30 *±* 5.06	33.87 *±* 6.26	34.06 *±* 7.00	34.43 *±* 6.13

ACA, adaptive conjoint analysis; CI, confidence interval; DM, decision making; SABR, stereotactic ablative radiotherapy; VCM, values clarification method.

a χ^2^(4) = 12.36, *P* = 0.015.

b Remaining diagnoses include gastric/liver cancer (1.1%), colorectal cancer (7.4%), gynecologic cancer (6.7%), blood/lymph cancer (3.2%), and “I don’t want to say” (0.4%).

c Remaining treatments include chemotherapy (8.9%), stereotactic ablative radiotherapy (1.1%), targeted therapy (0.4%), radiofrequency ablation (1.4%), immunotherapy (3.9%), other (8.9%), and “I don’t want treatment” (0.4%).

d *M_dif_* = 1.28, *P* = 0.012, 95% CI [0.22; 2.34].

e *M_dif_* = 1.07, *P* = 0.049, 95% CI [0.00; 2.15].

Exploratory linear regression analyses were performed to explore the relationship between health literacy and subjective numeracy and between health literacy and subjective numeracy on one hand and the primary and secondary outcomes on the other hand (Appendix E). Exploratory ANOVAs were performed to explore the interaction between the type of VCM (independent variable) and health literacy and subjective numeracy as moderators on primary and secondary outcomes. No covariates were included in the exploratory linear regression analyses. All findings with *P*≤ 0.05 were considered significant. The cutoffs for effect sizes were 0.01 to 0.05 = small, 0.06 to 0.13 = medium, and ≥0.14 = large.

In Appendix F, additional (exploratory) analyses with binary hypothetical treatment preference are provided.

### Funding

Financial support for this study was provided by the Dutch Cancer Society (VU2015-7930). The funding agreement ensured the authors’ independence in designing the study, interpreting data, and reporting.

## Results

### Effect of VCM Type on Decisional Conflict and Perceived Values Clarity (H1 and H2)

The type of VCM did not significantly influence overall decisional conflict, *F*(2, 278) = 2.39, *P* = 0.093, η_partial_^2^ = 0.02, or perceived values clarity specifically, *F*(2, 278) = 1.61, *P* = 0.20, η_partial_^2^ = 0.01. H1 and H2 were rejected.

### Moderation Effects of Age and Deliberation on Decisional Conflict and Perceived Values Clarity (RQ1 and RQ2)

**Figure 2:**
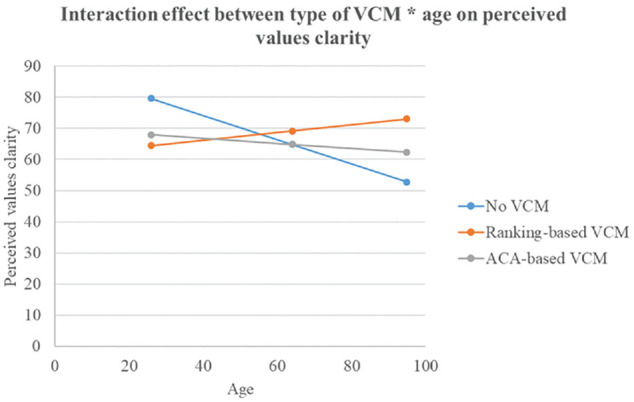
Interaction effect “Type of VCM × Age” on perceived values clarity. VCM, values clarification method. The *y*-axis represents the scores on the Values Clarity subscale of the Decisional Conflict Scale. Possible range scores: 0 = no values clarity to 100 = highest possible values clarity.

There was a significant interaction effect between type of VCM and age on perceived values clarity, *F*(2, 275) = 3.40, *P* = 0.035, η_partial_^2^ = 0.02. In younger participants, those exposed to no VCM experienced more values clarity than those exposed to the ranking-based VCM. In older participants, those exposed to the ranking-based VCM experienced more values clarity than those exposed to no VCM (see Figure 2). There was no significant interaction effect between type of VCM and age on overall decisional conflict, *F*(2, 275) = 1.88, *P* = 0.155, η_partial_^2^ = 0.01.

No significant interaction was found between the type of VCM and level of deliberation on overall decisional conflict, *F*(2, 275) = 2.30, *P* = 0.102, η_partial_^2^ = 0.02, or on perceived values clarity, *F*(2, 275) = 1.28, *P* = 0.22, η_partial_^2^ = 0.01.

### Effects of VCM Type on Secondary Outcomes (RQ3)

Type of VCM significantly affected hypothetical treatment preference, *F*(2, 278) = 4.52, *P =* 0.012, η_partial_^2^ = 0.03. Participants who had been exposed to the ACA-based VCM more often preferred SABR on average compared with those exposed to no VCM (*M_dif_* = −1.22, *P* = 0.018, 95% CI [−2.28; −0.16]) and those exposed to the ranking-based VCM (*M_dif_* = −1.08, *P* = 0.045, 95% CI [−2.15; −0.02]).

No significant main effect of type of VCM was found for any of the other secondary outcomes. Table 3 provides the *F*-test statistics of nonsignificant results.

**Table 3:** *F*-Test Statistics of Nonsignificant Results

	Effects of VCM Type on Secondary Outcomes (RQ3)	Moderation Effects of Age and Deliberation on Secondary Outcomes (RQ4 and RQ5)		Exploratory Analyses: Type of VCM × Health Literacy	Exploratory Analyses: Type of VCM × Subjective Numeracy
		Type of VCM × Age	Type of VCM × Deliberation		
Decisional conflict	x	x	x	*F*(2, 276) = 1.47, *P* = 0.23, η_partial_^2^ = 0.01	*F*(2, 276) = 6.21, *P* = 0.002, η_partial_^2^ = 0.04
Perceived values clarity	x	x	x	*F*(2, 276) = 0.64, *P* = 0.53, η_partial_^2^ = 0.01	*F*(2, 276) = 3.21, *P* = 0.042, η_partial_^2^ = 0.02
Perceived cognitive load	*F*(2, 278) = 0.35, *P =* 0.70, η_partial_^2^ = 0.00	*F*(2, 275) = 0.29, *P =* 0.75, η_partial_^2^ = 0.00	*F*(2, 275) = 2.82, *P* = 0.061, η_partial_^2^ = 0.02	*F*(2, 276) = 2.84, *P* = 0.060, η_partial_^2^ = 0.02	*F*(2, 276) = 4.54, *P* = 0.011, η_partial_^2^ = 0.03
Anticipated regret	*F*(2, 278) = 0.99, *P =* 0.37, η_partial_^2^ = 0.01	*F*(2, 275) = 0.80, *P =* 0.45, η_partial_^2^ = 0.01	*F*(2, 275) = 1.91, *P =* 0.150, η_partial_^2^ = 0.01	*F*(2, 276) = 0.88, *P* = 0.42, η_partial_^2^ = 0.01	*F*(2, 276) = 0.25, *P* = 0.78, η_partial_^2^ = 0.00
Ambivalence regarding surgery	*F*(2, 278) = 0.09, *P =* 0.91, η_partial_^2^ = 0.00	*F*(2, 275) = 1.25, *P =* 0.29, η_partial_^2^ = 0.01	*F*(2, 275) = 0.80, *P =* 0.45, η_partial_^2^ = 0.01	*F*(2, 276) = 1.16, *P* = 0.31, η_partial_^2^ = 0.01	*F*(2, 276) = 1.39, *P* = 0.25, η_partial_^2^ = 0.01
Ambivalence regarding SABR	*F*(2, 278) = 0.62, *P =* 0.54, η_partial_^2^ = 0.00	*F*(2, 275) = 1.69, *P =* 0.187, η_partial_^2^ = 0.01	*F*(2, 275) = 0.45, *P =* 0.64, η_partial_^2^ = 0.00	*F*(2, 276) = 2.17, *P* = 0.117, η_partial_^2^ = 0.02	*F*(2, 276) = 0.18, *P* = 0.83, η_partial_^2^ = 0.00
Preparedness for decision making	*F*(2, 278) = 1.41, *P =* 0.25, η_partial_^2^ = 0.01	*F*(2, 275) = 1.09, *P =* 0.34, η_partial_^2^ = 0.01	*F*(2, 275) = 2.79, *P =* 0.063, η_partial_^2^ = 0.02	*F*(2, 276) = 1.30, *P* = 0.27, η_partial_^2^ = 0.01	*F*(2, 276) = 1.72, *P* = 0.182, η_partial_^2^ = 0.01
Hypothetical treatment preference	*F*(2, 278) = 4.52, *P =* 0.012, η_partial_^2^ = 0.03	*F*(2, 275) = 0.68, *P =* 0.51, η_partial_^2^ = 0.01	*F*(2, 275) =2.65, *P =* 0.073, η_partial_^2^ = 0.02	*F*(2, 276) = 3.04, *P* = 0.049, η_partial_^2^ = 0.02	*F*(2, 276) = 2.00, *P* = 0.138, η_partial_^2^ = 0.01

SABR, stereotactic ablative radiotherapy; VCM, values clarification method.

### Moderation Effects of Age and Deliberation on Secondary Outcomes (RQ4 and RQ5)

No significant interaction effects were found between type of VCM and age or type of VCM and level of deliberation for any of the secondary outcomes (see Table 3).

### Exploratory Analyses: Type of VCM × Health Literacy

No significant interaction effects were found between type of VCM and health literacy for overall decisional conflict, perceived values clarity, or any of the other secondary outcomes (see Table 3). For hypothetical treatment preference, there seemed to be a significant interaction effect. However, the parameter estimates did not show any significant *P* values. This implies that the difference was significant between ranking-based VCM and ACA-based VCM. Hence, with “no VCM” as the reference group in analyses with health literacy as a continuous moderator, the analyses did not yield significant differences.

### Exploratory Analyses: Type of VCM × Subjective Numeracy

**Figure 3:**
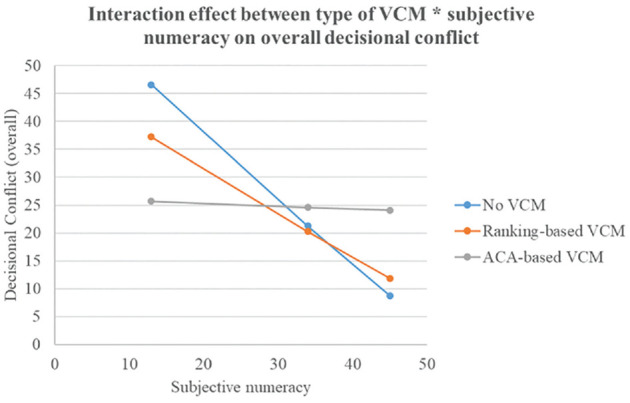
Interaction effect “Type of VCM × Subjective numeracy” on overall decisional conflict. VCM, values clarification method. The *y*-axis represents the scores on the total Decisional Conflict Scale. Possible range scores: 0 = no decisional conflict to 100 = highest possible decisional conflict.

The interaction between type of VCM and subjective numeracy was significant for overall decisional conflict, *F*(2, 276) = 6.21, *P* = 0.002, η_partial_^2^ = 0.04; perceived values clarity, *F*(2, 276) = 3.21, *P* = 0.042, η_partial_^2^ = 0.02; and perceived cognitive load, *F*(2, 276) = 4.54, *P* = 0.011, η_partial_^2^ = 0.03. For overall decisional conflict (see Figure 3), in lower numerate participants, those exposed to the ACA-based VCM experienced lower decisional conflict than those exposed to no VCM did. In higher numerate participants, however, those exposed to no VCM experienced lower decisional conflict than those exposed to the ACA-based VCM did.

**Figure 4:**
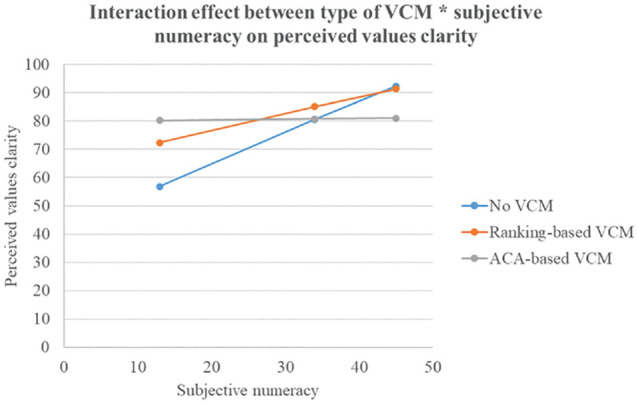
Interaction effect “Type of VCM × Subjective numeracy” on perceived values clarity. VCM, values clarification method. The *y*-axis represents the scores on the Values Clarity subscale of the Decisional Conflict Scale. Possible range scores: 0 = no values clarity to 100 = highest possible values clarity.

For perceived values clarity (see Figure 4), in lower numerate participants, those exposed to the ACA-based VCM experienced higher values clarity than those exposed to no VCM did. In higher numerate participants, however, those exposed to no VCM experienced higher values clarity than those exposed to the ACA-based VCM did.

**Figure 5:**
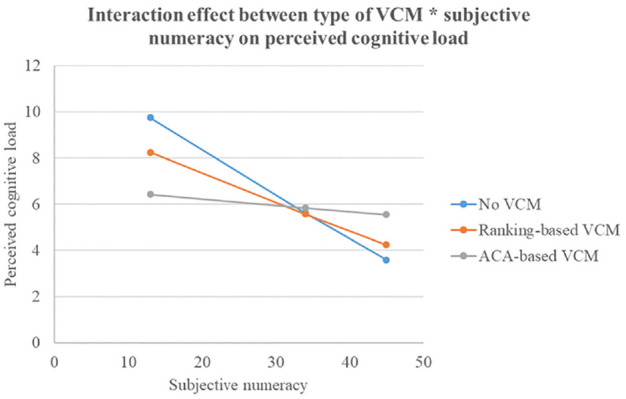
Interaction effect “Type of VCM × Subjective numeracy” on perceived cognitive load. VCM, values clarification method. The *y*-axis represents the scores on total perceived cognitive load. Possible range scores: 0 = no perceived cognitive load to 16 = highest possible cognitive load.

As for the secondary outcome of perceived cognitive overload (see Figure 5), in lower numerate participants, those exposed to the ACA*-*based VCM experienced lower cognitive load than those exposed to no VCM did. In higher numerate participants, however, those exposed to no VCM experienced lower cognitive load than those exposed to the ACA-based VCM did.

No significant interaction effects between type of VCM and subjective numeracy were found for any of the other secondary outcomes (see Table 3).

### Exploratory Analyses: Effects on Values Congruence

There was a significant association between type of VCM and values congruence (*P* = 0.022). Participants exposed to the *ACA*-based VCM had higher odds of reporting a values-congruent treatment preference than participants in the control condition (odds ratio [OR] = 0.42, *P* = 0.007, 95% CI [0.23; 0.79]) and participants exposed to the ranking-based VCM (OR = 0.52, *P* = 0.043, 95% CI [0.28; 0.98]). The odds of reporting a values-congruent preference was not significantly different between the control condition and the ranking-based VCM condition (*P* = 0.47). No significant moderation effects of age (*P* = 0.133), level of deliberation (*P* = 0.69), health literacy (*P* = 0.29), or subjective numeracy (*P* = 0.28) were found.

## Discussion

This study tested the effect of an ACA-based VCM in a PtDA about lung cancer treatment compared with a ranking-based VCM and no VCM on several decision-related outcomes among (treated) cancer patients. Contrary to our hypotheses, type of VCM did not affect patients’ experienced overall decisional conflict or perceived values clarity specifically. Age moderated the effect of type of VCM on perceived values clarity: younger participants experienced higher values clarity when exposed to no VCM (v. ranking-based VCM), while older participants experienced higher values clarity when exposed to the ranking-based VCM (v. no VCM). While no further effects were found on our secondary outcomes, except hypothetical treatment preference, notably, type of VCM did influence patients’ values congruence (assessed using a proxy).

Contrary to our hypotheses, the type of VCM did not influence decisional conflict or its subscale perceived values clarity, which constituted our primary outcomes. Exploratory subgroup analyses suggested that there may be negative consequences of using an ACA-based VCM (compared with no VCM) on decisional conflict, perceived values clarity, and perceived cognitive load among those with higher subjective numeracy. Several previous studies found an effect of an (A)CA-based VCM on decisional conflict but mostly when comparing scores to a pretest situation (e.g., de Achaval et al.^29^ and Hampson et al.^26^). For example, de Achaval et al.^29^ demonstrated a significant difference in decisional conflict before versus after intervention (i.e., ACA-based VCM). However, no significant differences between different types of VCMs were found in that study (i.e., ACA-based VCM, video booklet only, control group). Pignone et al.^25^ also did not find differences between a CA-based VCM and other VCM types on perceived values clarity. We expected that the feedback given after the task was easy to understand and would contribute directly to perceived values clarity. However, as de Achaval et al.^29^ also concluded, an ACA-based VCM may require more intensive cognitive involvement than we assumed, which may explain the lack of improvement in decisional conflict/perceived values clarity. It should also be noted that low decisional conflict and high perceived values clarity were already apparent in the control condition, so it is questionable to what extent further improvements were possible.

Interestingly, the ACA-based VCM, as compared with the other experimental conditions, resulted in higher values congruence. This result should be interpreted with caution since we used a proxy for values congruence that has some limitations (e.g., we measured treatment preference and not a final decision; we only used the number one priority excluding survival). However, the different effects found (i.e., higher values congruence in those exposed to the ACA-based VCM, higher perceived values clarity, lower overall decisional conflict, and lower perceived cognitive load in those exposed to the ACA-based VCM among lower numerate patients) suggest that the ACA-based VCM induced another type of reasoning. It could be that patients in our sample evaluated SABR more positively than surgery based on the prototype PtDA alone, possibly because SABR is associated with less severe impact on the shorter term. While completing the ACA-based VCM, the main benefit of surgery (i.e., more certainty about treatment success) may have been more evident to lower numerate patients. Previous experience with surgery among participants in the ACA-based condition did not explain the finding, as there were no significant between-condition differences in the proportion of participants who had undergone cancer surgery in the past, χ^2^(2, *N* = 282) = 3.89, *P* = 0.143.

The finding that the ACA-based VCM facilitated values-congruent preferences may imply that the ACA-based VCM indeed induced a more intuitive (v. analytic) treatment preference. The fact that this did not translate to higher perceived values clarity might be related to the phenomenon that when people think about their reasons to prefer certain options, they often use rationalizations in retrospect—rationalizations not used when they more intuitively/holistically evaluate options.^44^ Although we cannot conclude this for sure, it might be that values congruence more adequately reflected participants’“true” values clarity than our measure of “perceived values clarity.” Given the high scores on perceived values clarity across conditions, it is also possible that participants (mistakenly) felt that they knew what mattered to them in the decision merely because they had been exposed to information about the options in the prototype PtDA.

Finally, the interaction effects found between the type of VCM and subjective numeracy again demonstrate the importance of numeracy in considering treatment options, and therefore in shared decision making and PtDAs. This is in line with previous studies concluding that people with higher numeracy are better able to elicit consistent (perceived) values.^45–49^

### Limitations and Future Research

Our study has some limitations. First, we used a hypothetical scenario, and second, we included analog patients only (i.e., people asked to imagine the hypothetical situation of the target population). We did not measure the level of engagement with the topic. Although this has been shown to be a valid method,^50^ we acknowledge that it may have limited participant engagement. A previous study showed that decisional conflict scores, and improvements therein after exposure to decision support tools, are the largest when someone is ill and has to make a decision for oneself.^51^ Third, although we provided instant feedback about relative attribute importance, we did not show participants explicitly how the ACA task outcome aligned with the treatment options (i.e., “decision analysis”). This kind of feedback may be steering decisions too much. Nevertheless, previous studies have shown that this feedback is an important feature of more effective VCMs.^1^ Fourth (the wording of) some attributes in our PtDA may not always have been clear or too open to interpretation (e.g., “Almost full certainty about treatment success”). We did not perform a cognitive test to evaluate this. However, because the wording of attributes and levels was determined in consultation with medical professionals, patients, and researchers, we believe that comprehensibility was sufficient or at least representative for how treatment options are discussed in clinical practice. Further research is needed to unravel the unexpected finding that the ACA-based VCM affected treatment preference among patients who deliberated more, and more broadly how PtDAs elements work out for patients who differ in subjective numeracy and analytic/intuitive decision-making styles.

### Clinical Implications

It is difficult to formulate clear clinical implications, since our findings were mixed for different outcomes related to decision making. Completing an ACA-based VCM does not seem to outperform information only or a ranking-based VCM in the context of a hypothetical lung cancer treatment decision. For patients considering themselves more numerate in our experiment, this caused even more decisional conflict, less values clarity, and more cognitive load. On the other hand, the ACA-based VCM resulted in higher values congruence compared with information only. Further research in an actual patient population is needed to gain insight into the cognitive processes that different VCMs induce, including an investigation of effects on a more elaborate measure of values congruence.

## Conclusion

Overall, our study did not show beneficial effects of an ACA-based VCM to decrease decisional conflict in (treated) cancer patients or to increase perceived values clarity. However, the ACA-based VCM did result in higher values congruence. If a VCM were to be used, it is relevant to take patients’ age and level of subjective numeracy into account.

## Supplemental Material

sj-docx-1-mdm-10.1177_0272989X241298630 – Supplemental material for Use of Adaptive Conjoint Analysis–Based Values Clarification in a Patient Decision Aid Is Not Associated with Better Perceived Values Clarity or Reduced Decisional Conflict but Enhances Values Congruence Supplemental material, sj-docx-1-mdm-10.1177_0272989X241298630 for Use of Adaptive Conjoint Analysis–Based Values Clarification in a Patient Decision Aid Is Not Associated with Better Perceived Values Clarity or Reduced Decisional Conflict but Enhances Values Congruence by Nida Gizem Yılmaz, Arwen H. Pieterse, Danielle R. M. Timmermans, Annemarie Becker, Birgit Witte-Lissenberg and Olga C. Damman in Medical Decision Making

## Notes

i. For the sake of the current experiment, the chances of survival after treatment were presented as being equal (i.e., 70%). According to the National Institute for Health and Care Excellence (NICE) guideline, surgery is the standard of care in early-stage NSCLC, when the patient is fit. Stereotactic ablative radiotherapy (SABR) is recommended when the patient is not fit/when surgery is considered unsuitable/when the patient is not willing to accept surgery-related risks.^32^ Dalmia and colleagues^33^ have recently concluded that high-quality evidence comparing the effectiveness between surgery and SABR across clinical and patient-reported outcomes is not available and that most patients with early-stage NSCLC should therefore be involved in choosing between the options.
