# Supplementary material for: Use of Adaptive Conjoint Analysis–Based Values Clarification in a Patient Decision Aid Is Not Associated with Better Perceived Values Clarity or Reduced Decisional Conflict but Enhances Values Congruence
Source: Med Decis Making. 2024 Nov 18;45(1):109–23. doi: 10.1177/0272989X241298630 (PMC11645850; doi:10.1177/0272989X241298630)
Supplement: sj-docx-1-mdm-10.1177_0272989X241298630 – Supplemental material for Use of Adaptive Conjoint Analysis–Based Values Clarification in a Patient Decision Aid Is Not Associated with Better Perceived Values Clarity or Reduced Decisional Conflict but Enhances Values Congruence [file sj-docx-1-mdm-10.1177_0272989X241298630.docx]

**Appendices**

**Appendix A.** *Stimulus materials.*

**INTRODUCTORY INFORMATION (IDENTICAL ACROSS CONDITIONS)**

**You are going to view a patient decision aid. The patient decision aid concerns the treatment of early stage lung cancer, and can support patients in making a decision regarding their treatment.**

**Early stage non-small cell lung cancer**

The patient decision aid concerns early stage non-small cell lung cancer.

In early stage lung cancer, the tumor is located in the lung only. In non-small cell lung cancer, the tumor cells are quite big. Taking these two characteristics together, this type of lung cancer can be treated well.

**Treatment options**

*Surgery*

During a surgery, the tumor is being cut out. Surgery has always been the standard treatment option.

*Stereotactic ablative radiotherapy*

In this treatment, the tumor receives a high dose of radiation, which causes the tumor cells to die. The doctor irradiates very precisely only the tumor. Stereotactic ablative radiation is a newer treatment.

The chance of survival is the same for both treatments.

Are both treatments possible? Then, it is important to think carefully about your choice. Your preferences are important here.

**Assignment**

*Pretend* you have early stage non-small cell lung cancer. The doctor told you that you can opt for surgery or stereotactic ablative radiation. You will use the patient decision aid to make this choice.

View the patient decision aid now. When you have finished viewing part 1 of the patient decision aid, you can click on 'Continue'. Then part 2 of the patient decision aid follows. After part 2 you come to the questionnaire. You must indicate a preference for a treatment in the questionnaire. Remember you are *pretending*. You are not really opting for a treatment. Your answers and preferences have no consequences.

**PATIENT DECISION AID (IDENTICAL ACROSS CONDITIONS)**

**Patient decision aid – Part 1**

Below is an overview of the main differences between the two treatments. The overview was made by doctors and patients together.


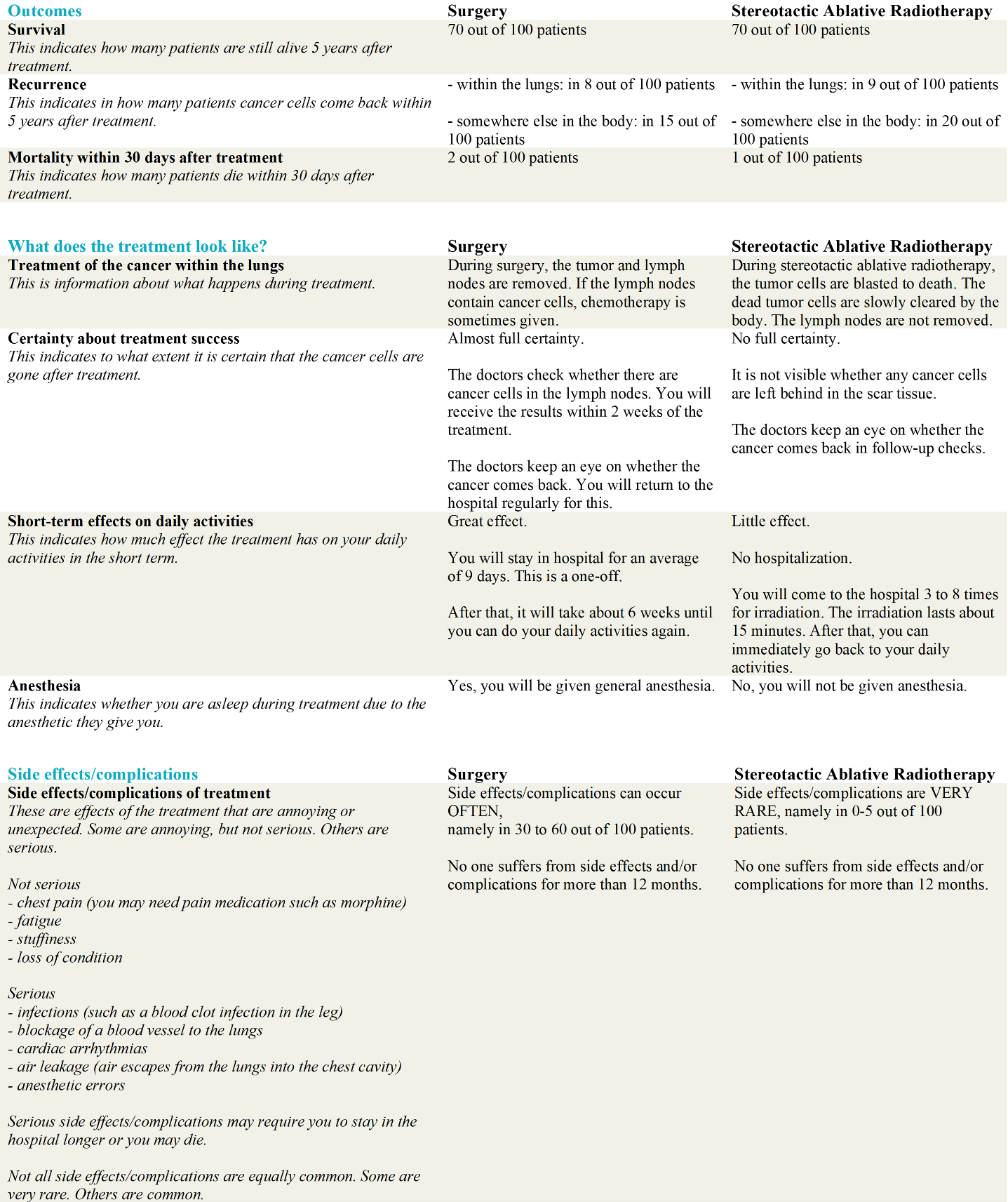


**VALUES CLARIFICATION (CONDITION: ‘*RANKING*-BASED VCM’)**

**Patient decision aid – Part 2**

You will do an exercise to determine what is important to you. This is part 2 of the patient decision aid.

You will soon see a number of consequences of the treatments. These consequences can play a role in which treatment you prefer. We ask you to indicate how important you think the various consequences are. You can put the consequences of treatments that are most important to you at the top. You can put the consequences of treatments that are least important to you at the bottom. You may drag the blocks.

| The chances of survival are high. |  |  |  |  |
| --- | --- | --- | --- | --- |
|  |  |  |  |  |
| Side effects/Complications are rare and mild. |  |  |  |  |
|  |  |  |  |  |
| Side effects/Complications are rare, but might be severe. |  |  |  |  |
|  |  | 🡪  🡨 |  |  |
| Side effects/Complications are  frequent, but mild. |  |  |  |  |
|  |  |  |  |  |
| There is almost full certainty about the success of the treatment. |  |  |  |  |
|  |  |  |  |  |
| On the short term, the treatment has little effect on daily activities. |  |  |  |  |

**VALUES CLARIFICATION (CONDITION: ‘*CONJOINT ANALYSIS*-BASED VCM’)**

**Patient decision aid – Part 2**

You will be referred to a short exercise to determine what is important to you. This is part 2 of the decision aid.


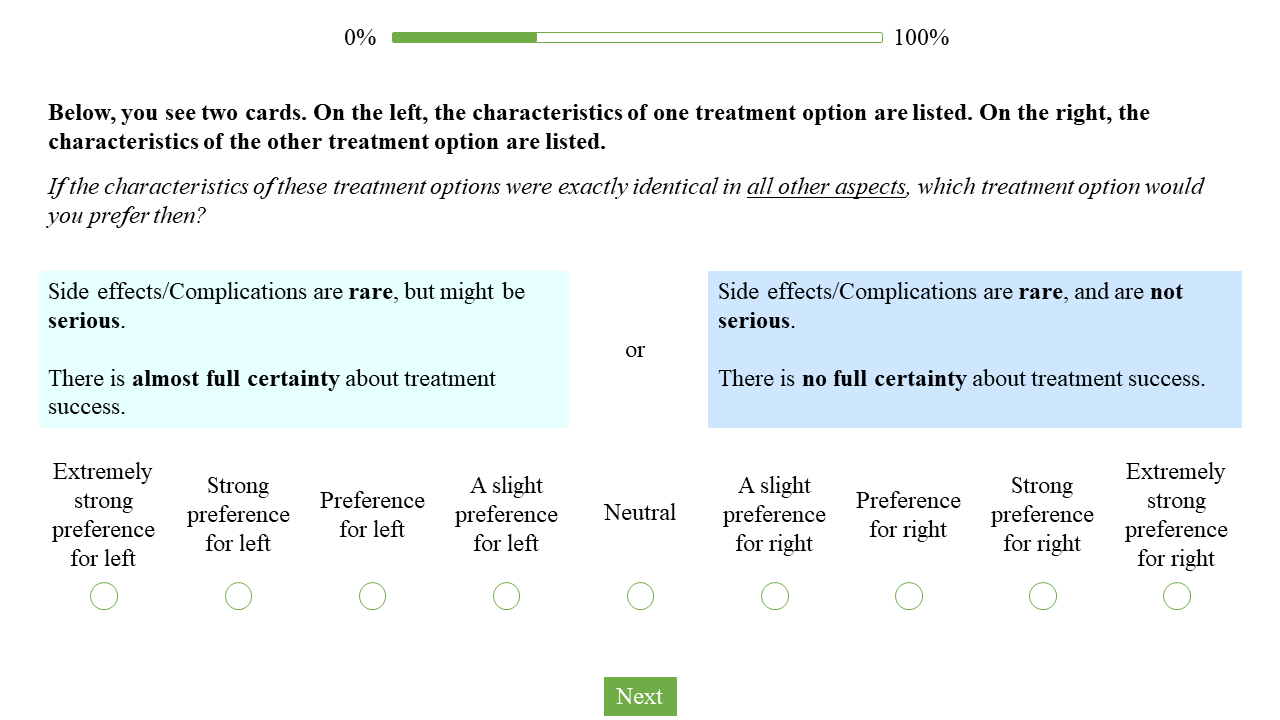


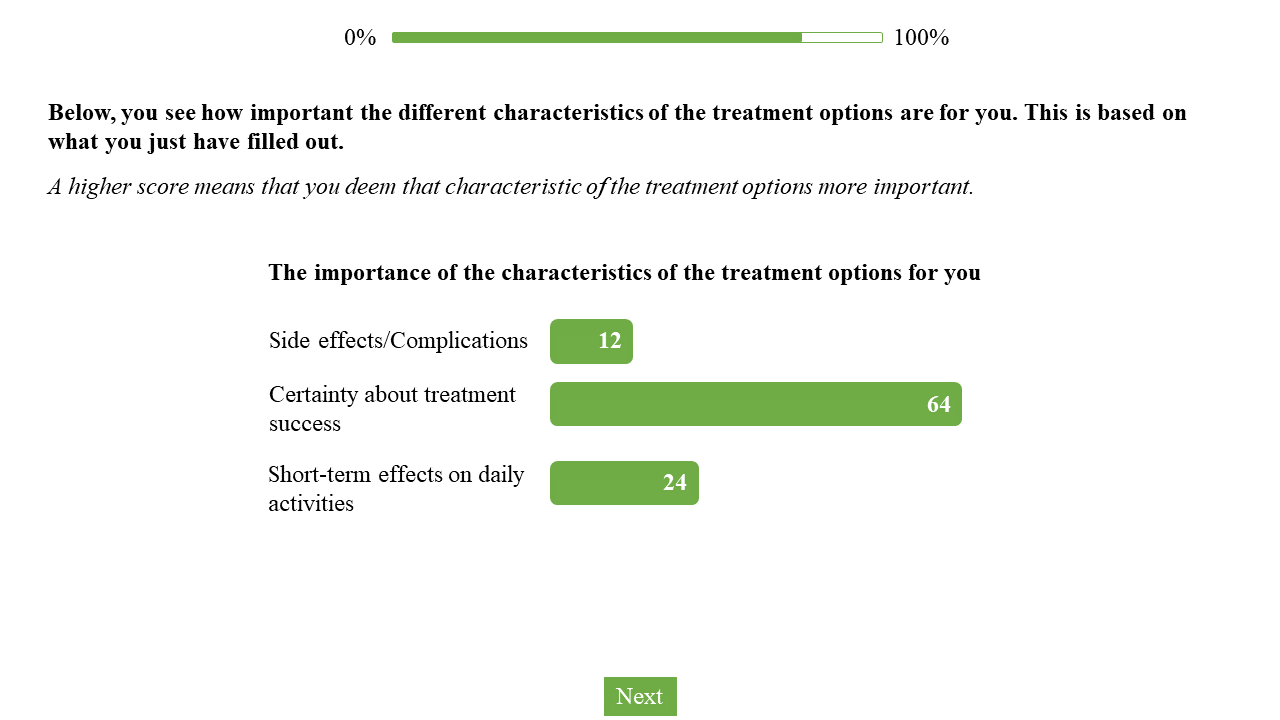


***NOTE:*** *The upper screenshot is one example out of the 12 pairs of vignettes. The bottom screenshot shows what the instant feedback to participants looked like.*

**Appendix B.** *Overview of the ranking of attributes across experimental conditions.*

The ranking of attributes in the *no VCM* condition can be found in *Table 1*. The ranking of attributes in the *ranking-based VCM* condition can be found in *Table 2*. The tables should be read horizontally. Per attribute, it is shown how many times (in %) the attribute was ranked first (#1) to last (#6) by participants, on average. Overall, “The chances of survival are high” was ranked as *most important* most often. However, as this attribute was kept constant in the current study, this ranking will be disregarded. This means that “There is almost full certainty about the success of the treatment” was perceived as most important, and that “Side effects/Complications are rare, but might be severe” and “On the short term, the treatment had little effect on daily activities” were perceived as least important. Other than that, “Side effects/Complications are rare and mild” was ranked second most important, and “Side effects/Complications are frequent, but mild” was ranked second least important.

**Table 1.** *Ranking of attributes in the no VCM condition.*

|  | **Ranking** | | | | | |
| --- | --- | --- | --- | --- | --- | --- |
| **Attribute** | **#1** | **#2** | **#3** | **#4** | **#5** | **#6** |
| The chances of survival are high | 67.7% | 15.2% | 6.1% | 4.0% | 4.0% | 3.0% |
| Side effects/Complications are rare and mild | 5.1% | 15.2% | **29.3%** | 24.2% | 17.2% | 9.1% |
| Side effects/Complications are rare, but might be severe | 6.1% | 5.1% | 20.2% | 9.1% | 26.3% | **33.3%** |
| Side effect/Complications are frequent, but mild | 1.0% | 4.0% | 12.1% | 29.3% | **35.4%** | 18.2% |
| There is almost full certainty about the success of the treatment | 15.2% | **49.5%** | 19.2% | 10.1% | 4.0% | 2.0% |
| On the short term, the treatment had little effect on daily activities | 5.1% | 11.1% | 13.1% | 23.2% | 13.1% | **34.3%** |

**Table 2.** *Ranking of attributes in the ranking-based VCM condition.*

|  | **Ranking** | | | | | |
| --- | --- | --- | --- | --- | --- | --- |
| **Attribute** | **#1** | **#2** | **#3** | **#4** | **#5** | **#6** |
| The chances of survival are high | 73.7% | 15.8% | 5.3% | 3.2% | - | 2.1% |
| Side effects/Complications are rare and mild | 5.3% | 10.5% | **37.9%** | 20.0% | 16.8% | 9.5% |
| Side effects/Complications are rare, but might be severe | 1.1% | 5.3% | 10.5% | 13.7% | 21.1% | **48.4%** |
| Side effect/Complications are frequent, but mild | 1.1% | 4.2% | 5.3% | 30.5% | **48.4%** | 10.5% |
| There is almost full certainty about the success of the treatment | 16.8% | **55.8%** | 15.8% | 8.4% | 1.1% | 2.1% |
| On the short term, the treatment had little effect on daily activities | 2.1% | 8.4% | 25.3% | 24.2% | 12.6% | **27.4%** |

The distribution of the importance of the levels of the attributes (i.e., partworth utilities) in the *conjoint analysis-based VCM* condition can be found in *Table 3*. Similar to the other two experimental conditions, “The chances of survival are high” was the most important attribute. The least important attribute was “Side effects/Complications are frequent, but mild”.

**Table 3.** *Distribution of the importance of attributes in the conjoint analysis-based*

*VCM condition.*

| **Attribute** | **Mean expected utility** |
| --- | --- |
| The chances of survival are high | 73.02 |
| There is almost full certainty about the success of the treatment | 48.57 |
| Side effects/Complications are rare, but might be severe | -39.84 |
| On the short term, the treatment had little effect on daily activities | 37.60 |
| Side effects/Complications are rare and mild | 33.06 |
| Side effect/Complications are frequent, but mild | 6.78 |

**Appendix C.** *Measures:* *the items used per variable.*

**DECISIONAL CONFLICT**

1. Do you know which options are available to you?
2. Do you know the benefits of each option?
3. Do you know the risks and side effects of each option?
4. Are you clear about which benefits matter most to you? *
5. Are you clear about which risks and side effects matter most to you?*
6. Are you clear about which is more important to you (the benefits or the risks and side effects? *
7. Do you have enough support from others to make a choice?
8. Are you choosing without pressure from others?
9. Do you have enough advice to make a choice?
10. Are you clear about the best choice for you?
11. Do you feel sure about what to choose?
12. Is this decision easy for you to make?
13. Do you feel you have made an informed decision?
14. Does your decision show what is important to you?
15. Do you expect to stick with your decision?
16. Are you satisfied with your decision?

* Items from the Values Clarity subscale

*Answer options: 0 = ‘yes’ to 4 = ‘no’*

**PERCEIVED COGNITIVE LOAD**

1. I had difficulty understanding how the information was structured into a coherent story.
2. Sometimes I felt “lost” when reading the story.
3. The main point of the story were clear and coherent. (reversed)
4. It was clear how all the information fit into the story as a whole. (reversed)

*Answer options: 1 = ‘strongly disagree’ to 7 = ‘strongly agree’*

**PREPAREDNESS FOR DECISION-MAKING**

Did this information:

1. Help you recognize that a decision needs to be made?
2. Help you to make a decision?
3. Help you think about the pros and cons of the treatment options?
4. Help you think about which pros and cons are most important to you?
5. Help you know that the treatment decision depends on what matters most to you?
6. Help you organize your own thoughts about the decision?

*Answer options: 0 = ‘not at all’ to 4 = ‘a great deal’*

**AMBIVALENCE**

1. Think about **surgery**. Considering your **positive thoughts and feelings** only and ignoring your negative thoughts and feelings about **surgery**, how **positive** are you about **surgery**?
2. Think about **surgery**. Considering your **negative thoughts and feelings** only and ignoring your positive thoughts and feelings about **surgery**, how **negative** are you about **surgery**?
3. Think about **stereotactic ablative radiotherapy**. Considering your **positive thoughts and feelings** only and ignoring your negative thoughts and feelings about **stereotactic ablative radiotherapy**, how **positive** are you about **stereotactic ablative radiotherapy**?
4. Think about **stereotactic ablative radiotherapy**. Considering your **negative thoughts and feelings** only and ignoring your positive thoughts and feelings about **stereotactic ablative radiotherapy**, how **negative** are you about **stereotactic ablative radiotherapy**?

*Answer options: 0 = ‘not positive/negative at all’ to 3 = ‘very positive/negative’*

**DELIBERATION**

1. I have tried to mentally visualize that I have to choose a treatment for early stage non-small cell lung cancer.
2. I have imagined how I would feel if I had surgery.
3. I have imagined how I would feel if I received stereotactic ablative radiotherapy.
4. I have tried to think through the consequences of having a surgery.
5. I have tried to think through the consequences of receiving stereotactic ablative radiotherapy.
6. I have made a (mental) list of the pros and cons of having a surgery.
7. I have made a (mental) list of the pros and cons of receiving stereotactic ablative radiotherapy.

*Answer options: 0 = ‘totally disagree’ to 4 = ‘totally agree’*

**HEALTH LITERACY**

1. How often do you have someone help you read hospital materials?
2. How confident are you filling out forms by yourself?
3. How often do you have problems learning about your medical condition because of difficulty reading hospital materials?

*Answer options: 1 = ‘never’ to 5 = ‘always’*

**SUBJECTIVE NUMERACY**

1. How good are you at working with fractions?
2. How good are you at working with percentages?
3. How good are you at calculating a 15% tip?
4. How good are you at figuring out how much a shirt will cost if it is 25% off?

*Answer options:* *1= ‘not at all good’ to 6 = ‘extremely good’*

1. When reading the newspaper, how helpful do you find tables and graphs that are parts of a story?

*Answer options: 1 = ‘not at all helpful’ to 6 = ‘extremely helpful’*

1. When people tell you the chance of something happening, do you prefer that they use words ("it rarely happens") or numbers ("there's a 1% chance")?

*Answer options: 1 = ‘always prefer words’ to 6 = ‘always prefer numbers’*

1. When you hear a weather forecast, do you prefer predictions using percentages (e.g., “there will be a 20% chance of rain today”) or predictions using only words (e.g., “there is a small chance of rain today”)?

*Answer options: 1 = ‘always prefer percentages’ to 6 = ‘always prefer words’*

1. How often do you find numerical information to be useful?

*Answer options: 1 = ‘never’ to ‘very often’*

**Appendix D.** *Process of constructing a proxy for values congruence.*

For all participants, first, an “expected treatment preference” was constructed. In the *no VCM* and *ranking*-based VCM conditions, the most important attribute (i.e., number 1 in the ranking) was considered to represent the expected treatment preference. If attribute *“The chances of survival are high”* (i.e., the attribute that was kept identical across conditions in the current study) was ranked first, the second most important attribute (i.e., number 2 in the ranking) was considered to represent the expected treatment preference. Indeed, attribute *“The chances of survival are high”* did not discriminate between SABR and surgery. The most fitting treatment option to each attribute, and the rationale for the correspondence, is shown in the table below.

As can be seen in *Table 1*, all attributes except attribute 5 (i.e., *“There is almost full certainty about the success of the treatment”*) indicated that SABR should be the most values congruent choice. If attribute 5 was ranked highest or second-highest, this indicated that surgery would be the most values congruent choice. All other attributes ranked at place #1 or #2 indicated that SABR would be the most values congruent choice. Hence, even though the second most important attribute might possibly not be the one that should get 100% weight, looking beyond the second most important attribute would not have yielded different conclusions in terms of most fitting (i.e., expected) treatment preference. Following the same line of argumentation, the attribute with the highest expected utility score in the conjoint analysis-based VCM condition was considered to represent the expected treatment preference.

Once an expected treatment preference was constructed for all participants, a new variable representing values congruence was constructed. If the expected and hypothetical treatment preference were aligned, this was labelled as “values congruence (1)”. If the expected and hypothetical treatment preference were not aligned, this was labelled as “values incongruence (0)”.

**Table 1.** *Attributes’ representation of treatment options.*

| **Attribute** | **Most fitting choice** | **Explanation** |
| --- | --- | --- |
| 1 = The chances of survival are high. | Not applicable | This attribute was kept constant in the current study, hence, its ranking was disregarded in further analyses. |
| 2 = Side effects/Complications are rare and mild. | SABR | SABR rarely causes side effects. |
| 3 = Side effects/Complications are rare, but might be severe. | SABR | SABR rarely causes side effects. Besides, “might be severe” refers to complications, which can occur after surgery, not after SABR. |
| 4 = Side effect/Complications are frequent, but mild. | SABR | Side effects and complications are more frequent after surgery. |
| 5 = There is almost full certainty about the success of the treatment. | Surgery | After surgery, there is more certainty about treatment success compared to after SABR. |
| 6 = On the short term, the treatment had little effect on daily activities. | SABR | On the short term, SABR has less effect on daily activities than surgery. |

**Appendix E.** *Results of exploratory analyses regarding the relationship between health literacy and subjective numeracy, and the primary and secondary outcomes.*

*Exploratory analyses regarding health literacy*

A higher level of health literacy appeared to be associated with a lower score on overall decisional conflict, *β*=-1.94, *t(280)*=-4.58, *p*<.000, 95% CI [-2.78;-1.11], as well as on anticipated regret, *β*=-0.25, *t(280)*=-3.98, *p*<.000, 95% CI [-0.38;-0.13] and perceived cognitive load, *β*=-0.41, *t(280)*=-5.99, *p*<.000, 95% CI [-0.54;-0.28]. A higher level of health literacy was also associated with a higher score on perceived values clarity, *β*=1.61, *t(280)*=2.99, *p*=.003, 95% CI [0.55;2.66], and preparedness for decision-making, *β*=0.59, *t(280)*=5.47, *p*< .000, 95% CI [0.38;0.80].

Health literacy was not associated with ambivalence regarding surgery, *β*=-0.02, *t(280)*=-0.70, *p*=.484, 95% CI [-0.08;0.04]), ambivalence regarding SABR, *β*=-0.04, *t(280)*=-1.31, *p=*.191, 95% CI [-0.10;0.02]), nor hypothetical treatment preference, *β*=0.08, *t(280)*=0.85, *p*=.396, 95% CI [-0.11;0.26].

*Exploratory analyses regarding subjective numeracy*

A higher level of subjective numeracy was associated with a lower score on overall decisional conflict, *β*=-0.59, *t(280)*=-4.41, *p*<.000, 95% CI [-0.86;-0.33], as well as on anticipated regret, *β*=-0.07, *t(280)*=-3.28, *p*=.001, 95% CI [-0.11;-0.03] and perceived cognitive load, *β*=-0.10, *t(280)*=-4.61, *p*<.000, 95% CI [-0.15;-0.06]. A higher level of subjective numeracy was associated with a higher score on perceived values clarity, *β*=0.47, *t(280)*=2.74, *p*=.006, 95% CI [0.13; 0.80], and preparedness for decision-making, *β*=0.19, *t(280)*=5.51, *p*<.000, 95% CI [0.12;0.25].

Subjective numeracy was not significantly associated with ambivalence regarding surgery, *β*=-0.00, *t(280)*=-0.12, *p*=.909, 95% CI [-0.02;0.02], ambivalence regarding SABR, *β*=0.01, *t(280)*=1.10, *p*=.272, 95% CI [-0.01;0.03], nor with hypothetical treatment preference, *β*=0.06, *t(280)*=1.92, *p*=.056, 95% CI [-0.00;-0.12].

**Appendix F.** *Additional (exploratory) analyses for hypothetical treatment preference (dichotomous).*

The original “hypothetical treatment preference” variable was measured on a 10-point scale (1 = strong preference for surgery to 10 = strong preference for SABR). A score of 5.5 meant that the participant was neutral; a score below 5.5 indicated a preference towards surgery, and a score above 5.5 indicated a preference towards SABR. Two participants scored exactly 5.5 and were excluded from the analyses with the dichotomized variable.

*Effects of VCM type on secondary outcomes (RQ3)*

A logistic regression was performed to ascertain the effects of type of VCM on the likelihood that patients would choose SABR over surgery. The logistic regression model was statistically significant, *χ^2^(3, N=280)*=13.42, *p*=.004. The model explained 6.3% (Nagelkerke R^2^) of the variance in hypothetical treatment preference and correctly classified 93.8% of cases. After adjusting for subjective numeracy, there was a significant association between type of VCM and hypothetical treatment preference (*p*=.007). The participants who had been exposed to the *conjoint analysis*-based VCM had 0.39 higher odds to choose SABR (vs surgery) compared to the participants who had not been exposed to a VCM (*OR*=0.39, *p*=.002, 95%CI [0.21 ; 0.71]). The odds of the participants who had been exposed to the *ranking*-based VCM were not significantly different than those of the participants who had not been exposed to a VCM (*p*=.329).

*Moderation effects of age and deliberation on secondary outcomes (RQ4 and RQ5)*

A logistic regression was performed to ascertain the interaction effects of type of VCM and age, and type of VCM and deliberation on the likelihood that patients would choose SABR over surgery. For the interaction effect with age, the logistic regression model was statistically significant, *χ^2^(6, N=280)*=17.25, *p*=.008. The model explained 8.0% (Nagelkerke R^2^) of the variance in hypothetical treatment preference and correctly classified 78.4% of cases. After adjusting for subjective numeracy, there was no significant interaction effect of type of VCM and age on hypothetical treatment preference (*p*=.582).

For the interaction effect with deliberation, the logistic regression model was statistically significant, *χ^2^(6, N=280)*=20.01, *p*=.003. The model explained 9.3% (Nagelkerke R^2^) of the variance in hypothetical treatment preference and correctly classified 82.7% of cases. After adjusting for subjective numeracy, there was no significant interaction effect of type of VCM and deliberation on hypothetical treatment preference (*p*=.164).

*Exploratory analyses: type of VCM * health literacy*

A logistic regression was performed to ascertain the interaction effects of type of VCM and health literacy on the likelihood that patients would choose SABR over surgery. The logistic regression model was statistically significant, *χ^2^(6, N=280)*=15.13, *p*=.019. The model explained 7.1% (Nagelkerke R^2^) of the variance in hypothetical treatment preference and correctly classified 87.7% of cases. After adjusting for subjective numeracy, there was no significant interaction effect of type of VCM and health literacy on hypothetical treatment preference (*p*=.465).

*Exploratory analyses: type of VCM * subjective numeracy*

A logistic regression was performed to ascertain the interaction effects of type of VCM and subjective numeracy on the likelihood that patients would choose SABR over surgery. The logistic regression model was statistically significant, *χ^2^(5, N=280)*=23.43, *p*<.001. The model explained 10.8% (Nagelkerke R^2^) of the variance in hypothetical treatment preference and correctly classified 93.8% of cases. After adjusting for subjective numeracy, there was a significant interaction effect of type of VCM and subjective numeracy on hypothetical treatment preference (*p*=.009). In patients with low subjective numeracy, patients who were exposed to the *conjoint analysis*-based VCM had 0.15 higher odds to choose for SABR (vs surgery) than patients who had not received any VCM (*OR*=0.15, *p*=.002, 95%CI [0.06 ; 0.39]). Patients who were exposed to the ranking-based VCM had no significantly different odds than patients who had not received any VCM (*p*=.452). In patients with high subjective numeracy, there was no significant interaction effect (*p*=.748).
